# Higher longitudinal brain white matter atrophy rate in aquaporin-4 IgG-positive NMOSD compared with healthy controls

**DOI:** 10.1038/s41598-023-38893-1

**Published:** 2023-08-03

**Authors:** Hiroki Masuda, Masahiro Mori, Shigeki Hirano, Akiyuki Uzawa, Tomohiko Uchida, Mayumi Muto, Ryohei Ohtani, Reiji Aoki, Yoshiyuki Hirano, Japanese Alzheimer’s Disease Neuroimaging Initiative (J-ADNI), Takeshi Iwatsubo, Takashi Asada, Hiroyuki Arai, Morihiro Sugishita, Hiroshi Matsuda, Kengo Ito, Michio Senda, Kenji Ishii, Ryozo Kuwano, Takeshi Ikeuchi, Noriko Sato, Hajime Sato, Shun Shimohama, Masaki Saitoh, Rika Yamauchi, Takashi Hayashi, Seiju Kobayashi, Norihito Nakano, Junichiro Kanazawa, Takeshi Ando, Chiyoko Takanami, Masato Hareyama, Masamitsu Hatakenaka, Eriko Tsukamoto, Shinji Ochi, Mikio Shoji, Etsuro Matsubara, Takeshi Kawarabayashi, Yasuhito Wakasaya, Takashi Nakata, Naoko Nakahata, Shuichi Ono, Yoshihiro Takai, Satoshi Takahashi, Hisashi Yonezawa, Junko Takahashi, Masako Kudoh, Makoto Sasaki, Yutaka Matsumura, Yohsuke Hirata, Tsuyoshi Metoki, Susumu Hayakawa, Yuichi Sato, Masayuki Takeda, Toshiaki Sasaki, Koichiro Sera, Kazunori Terasaki, Yoshihiro Saitoh, Shoko Goto, Kuniko Ueno, Hiromi Sakashita, Kuniko Watanabe, Ken Nagata, Yuichi Sato, Tetsuya Maeda, Yasushi Kondoh, Takashi Yamazaki, Daiki Takano, Mio Miyata, Hiromi Komatsu, Mayumi Watanabe, Tomomi Sinoda, Rena Muraoka, Kayoko Kikuchi, Hitomi Ito, Aki Sato, Toshibumi Kinoshita, Hideyo Toyoshima, Kaoru Sato, Shigeki Sugawara, Isao Ito, Fumiko Kumagai, Katsutoshi Furukawa, Masaaki Waragai, Naoki Tomita, Nobuyuki Okamura, Mari Ootsuki, Katsumi Sugawara, Satomi Sugawara, Shunji Mugikura, Atsushi Umetsu, Takanori Murata, Tatsuo Nagasaka, Yukitsuka Kudo, Manabu Tashiro, Shoichi Watanuki, Masatoyo Nishizawa, Takayoshi Tokutake, Saeri Ishikawa, Emiko Kishida, Nozomi Sato, Mieko Hagiwara, Kumi Yamanaka, Takeyuki Watanabe, Taeko Takasugi, Shoichi Inagawa, Kenichi Naito, Masanori Awaji, Tsutomu Kanazawa, Kouiti Okamoto, Masaki Ikeda, Tsuneo Yamazaki, Yuiti Tasiro, Syunn Nagamine, Shiori Katsuyama, Sathiko Kurose, Sayuri Fukushima, Etsuko Koya, Makoto Amanuma, Noboru Oriuti, Kouiti Ujita, Kazuhiro Kishi, Kazuhisa Tuda, Katsuyoshi Mizukami, Tetsuaki Arai, Etsuko Nakajima, Katsumi Miyamoto, Kousaku Saotome, Tomoya Kobayashi, Saori Itoya, Jun Ookubo, Toshiya Akatsu, Yoshiko Anzai, Junya Ikegaki, Yuuichi Katou, Kaori Kimura, Ryou Kuchii, Hajime Saitou, Kazuya Shinoda, Satoka Someya, Hiroko Taguchi, Kazuya Tashiro, Masaya Tanaka, Tatsuya Nemoto, Ryou Wakabayashi, Daisuke Watanabe, Harumasa Takano, Tetsuya Suhara, Hitoshi Shinoto, Hitoshi Shimada, Makoto Higuchi, Takaaki Mori, Hiroshi Ito, Takayuki Obata, Yoshiko Fukushima, Kazuko Suzuki, Izumi Izumida, Katsuyuki Tanimoto, Takahiro Shiraishi, Junko Shiba, Hiroaki Yano, Miki Satake, Aimi Nakui, Yae Ebihara, Tomomi Hasegawa, Yasumasa Yoshiyama, Mami Kato, Yuki Ogata, Hiroyuki Fujikawa, Nobuo Araki, Yoshihiko Nakazato, Takahiro Sasaki, Tomokazu Shimadu, Kimiko Yoshimaru, Hiroshi Matsuda, Etsuko Imabayashi, Asako Yasuda, Etuko Yamamoto, Natsumi Nakamata, Noriko Miyauchi, Keiko Ozawa, Rieko Hashimoto, Taishi Unezawa, Takafumi Ichikawa, Hiroki Hayashi, Masakazu Yamagishi, Tunemichi Mihara, Masaya Hirano, Shinichi Watanabe, Junichiro Fukuhara, Hajime Matsudo, Nobuyuki Saito, Atsushi Iwata, Hisatomo Kowa, Toshihiro Hayashi, Ryoko Ihara, Toji Miyagawa, Mizuho Yoshida, Yuri Koide, Eriko Samura, Kurumi Fujii, Kaori Watanabe, Nagae Orihara, Toshimitsu Momose, Akira Kunimatsu, Harushi Mori, Miwako Takahashi, Takuya Arai, Yoshiki Kojima, Masami Goto, Takeo Sarashina, Syuichi Uzuki, Seiji Katou, Yoshiharu Sekine, Yukihiro Takauchi, Chiine Kagami, Kazutomi Kanemaru, Shigeo Murayama, Yasushi Nishina, Maria Sakaibara, Yumiko Okazaki, Rieko Okada, Maki Obata, Yuko Iwata, Mizuho Minami, Yasuko Hanabusa, Hanae Shingyouji, Kyoko Tottori, Aya Tokumaru, Makoto Ichinose, Kazuya Kume, Syunsuke Kahashi, Kunimasa Arima, Tadashi Tukamoto, Shin Tanaka, Yuko Nagahusa, Masuhiro Sakata, Mitsutoshi Okazaki, Yuko Saito, Maki Yamada, Tiine Kodama, Maki Obata, Tomoko Takeuchi, Keiichiro Ozawa, Yuko Iwata, Hanae Shingyouji, Yasuko Hanabusa, Yoshiko Kawaji, Kyouko Tottori, Noriko Sato, Yasuhiro Nakata, Satoshi Sawada, Makoto Mimatsu, Daisuke Nakkamura, Takeshi Tamaru, Shunichirou Horiuchi, Heii Arai, Tsuneyoshi Ota, Aiko Kodaka, Yuko Tagata, Tomoko Nakada, Eizo Iseki, Kiyoshi Sato, Hiroshige Fujishiro, Norio Murayama, Masaru Suzuki, Satoshi Kimura, Masanobu Takahashi, Haruo Hanyu, Hirofumi Sakurai, Takahiko Umahara, Hidekazu Kanetaka, Kaori Arashino, Mikako Murakami, Ai Kito, Seiko Miyagi, Kaori Doi, Kazuyoshi Sasaki, Mineo Yamazaki, Akiko Ishiwata, Yasushi Arai, Akane Nogami, Sumiko Fukuda, Kyouko Tottori, Mizuho Minami, Yuko Iwata, Koichi Kozaki, Yukiko Yamada, Sayaka Kimura, Ayako Machida, Kuninori Kobayashi, Hidehiro Mizusawa, Nobuo Sanjo, Mutsufusa Watanabe, Takuya Ohkubo, Hiromi Utashiro, Yukiko Matsumoto, Kumiko Hagiya, Yoshiko Miyama, Takako Shinozaki, Haruko Hiraki, Hitoshi Shibuya, Isamu Ohashi, Akira Toriihara, Shinichi Ohtani, Toshifumi Matsui, Yu Hayasaka, Tomomi Toyama, Hideki Sakurai, Kumiko Sugiura, Hirofumi Taguchi, Shizuo Hatashita, Akari Imuta, Akiko Matsudo, Daichi Wakebe, Hideki Hayakawa, Mitsuhiro Ono, Takayoshi Ohara, Yukihiko Washimi, Yutaka Arahata, Akinori Takeda, Yoko Konagaya, Akiko Yamaoka, Masashi Tsujimoto, Hideyuki Hattori, Takashi Sakurai, Miura Hisayuki, Hidetoshi Endou, Syousuke Satake, Young Jae Hong, Katsunari Iwai, Kenji Yoshiyama, Masaki Suenaga, Sumiko Morita, Teruhiko Kachi, Kenji Toba, Rina Miura, Takiko Kawai, Ai Honda, Takashi Kato, Ken Fujiwara, Rikio Katou, Mariko Koyama, Naohiko Fukaya, Akira Tsuji, Hitomi Shimizu, Hiroyuki Fujisawa, Tomoko Nakazawa, Satoshi Koyama, Takanori Sakata, Masahito Yamada, Mitsuhiro Yoshita, Miharu Samuraki, Kenjiro Ono, Moeko Shinohara, Yuki Soshi, Kozue Niwa, Chiaki Doumoto, Mariko Hata, Miyuki Matsushita, Mai Tsukiyama, Nozomi Takeda, Sachiko Yonezawa, Ichiro Matsunari, Osamu Matsui, Fumiaki Ueda, Yasuji Ryu, Masanobu Sakamoto, Yasuomi Ouchi, Madoka Chita, Yumiko Fujita, Rika Majima, Hiromi Tsubota, Umeo Shirasawa, Masashi Sugimori, Wataru Ariya, Yuuzou Hagiwara, Yasuo Tanizaki, Hidenao Fukuyama, Ryosuke Takahashi, Hajime Takechi, Chihiro Namiki, Kengo Uemura, Takeshi Kihara, Hiroshi Yamauchi, Shizuko Tanaka-Urayama, Emiko Maeda, Natsu Saito, Shiho Satomi, Konomi Kabata, Shin-Ichi Urayama, Tomohisa Okada, Koichi Ishizu, Shigeto Kawase, Satoshi Fukumoto, Masanori Nakagawa, Takahiko Tokuda, Masaki Kondo, Fumitoshi Niwa, Toshiki Mizuno, Yoko Oishi, Mariko Yamazaki, Daisuke Yamaguchi, Kyoko Ito, Yoku Asano, Chizuru Hamaguchi, Kei Yamada, Chio Okuyama, Kentaro Akazawa, Shigenori Matsushima, Takamasa Matsuo, Toshiaki Nakagawa, Takeshi Nii, Takuji Nishida, Kuniaki Kiuchi, Masami Fukusumi, Hideyuki Watanabe, Toshiaki Taoka, Akihiro Nogi, Masatoshi Takeda, Toshihisa Tanaka, Naoyuki Sato, Hiroaki Kazui, Kenji Yoshiyama, Takashi Kudo, Masayasu Okochi, Takashi Morihara, Shinji Tagami, Noriyuki Hayashi, Masahiko Takaya, Tamiki Wada, Mikiko Yokokoji, Hiromichi Sugiyama, Daisuke Yamamoto, Shuko Takeda, Keiko Nomura, Mutsumi Tomioka, Eiichi Uchida, Yoshiyuki Ikeda, Mineto Murakami, Takami Miki, Hiroyuki Shimada, Suzuka Ataka, Motokatsu Kanemoto, Jun Takeuchi, Akitoshi Takeda, Rie Azuma, Yuki Iwamoto, Naomi Tagawa, Junko Masao, Yuka Matsumoto, Yuko Kikukawa, Hisako Fujii, Junko Matsumura, Susumu Shiomi, Joji Kawabe, Yoshihiro Shimonishi, Yukio Miki, Mitsuji Higashida, Tomohiro Sahara, Takashi Yamanaga, Shinichi Sakamoto, Hiroyuki Tsushima, Kiyoshi Maeda, Yasuji Yamamoto, Toshio Kawamata, Kazuo Sakai, Haruhiko Oda, Takashi Sakurai, Taichi Akisaki, Mizuho Adachi, Masako Kuranaga, Sachi Takegawa, Yoshihiko Tahara, Seishi Terada, Takeshi Ishihara, Hajime Honda, Osamu Yokota, Yuki Kishimoto, Naoya Takeda, Nao Imai, Mayumi Yabe, Kentaro Ida, Daigo Anami, Seiji Inoue, Toshi Matsushita, Reiko Wada, Shinsuke Hiramatsu, Hiromi Tonbara, Reiko Yamamoto, Kenji Nakashima, Kenji Wada-Isoe, Saori Yamasaki, Eijiro Yamashita, Yu Nakamura, Ichiro Ishikawa, Sonoko Danjo, Tomomi Shinohara, Miyuki Ueno, Yuka Kashimoto, Yoshihiro Nishiyama, Yuka Yamamoto, Narihide Kimura, Kazuo Ogawa, Yasuhiro Sasakawa, Takashi Ishimori, Yukito Maeda, Tatsuo Yamada, Shinji Ouma, Aika Fukuhara-Kaneumi, Nami Sakamoto, Rie Nagao, Kengo Yoshimitsu, Yasuo Kuwabara, Ryuji Nakamuta, Minoru Tanaka, Manabu Ikeda, Mamoru Hashimoto, Keiichirou Kaneda, Yuusuke Yatabe, Kazuki Honda, Naoko Ichimi, Fumi Akatuka, Mariko Morinaga, Miyako Noda, Mika Kitajima, Toshinori Hirai, Shinya Shiraishi, Naoji Amano, Shinsuke Washizuka, Toru Takahashi, Shin Inuzuka, Tetsuya Hagiwara, Nobuhiro Sugiyama, Yatsuka Okada, Tomomi Ogihara, Takehiko Yasaki, Minori Kitayama, Tomonori Owa, Akiko Ryokawa, Rie Takeuchi, Satoe Goto, Keiko Yamauchi, Mie Ito, Tomoki Kaneko, Hitoshi Ueda, Shuichi Ikeda, Masaki Takao, Ban Mihara, Hirofumi Kubo, Akiko Takano, Gou Yasui, Masami Akuzawa, Kaori Yamaguchi, Toshinari Odawara, Megumi Shimamura, Mikiko Sugiyama, Atsushi Watanabe, Naomi Oota, Shigeo Takebayashi, Yoshigazu Hayakawa, Mitsuhiro Idegawa, Noriko Toya, Kazunari Ishii, Satoshi Kuwabara

**Affiliations:** 1 Department of Neurology, Graduate School of Medicine, Chiba University, 1-8-1, Inohana, Chuo-Ku, Chiba, 260-8670 Japan; 2 Department of Neurology, Chiba Rosai Hospital, 2-16, Tatsumidai-Higashi, Ichihara, 290-0003 Japan; 3 Department of Neurology, Kimitsu Chuo Hospital, 1010, Sakurai, Kisarazu-Shi, Chiba, 292-8535 Japan; 4 Research Center for Child Mental Development, Chiba University, 1-8-1, Inohana, Chuo-Ku, Chiba, 260-8670 Japan; 5 University of Tokyo, Bunkyo-Ku, Japan; 6 Tsukuba University Hospital, Tsukuba-Shi, Japan; 7 Tohoku University Hospital, Sendai-Shi, Japan; 8 Research Institute for Brain and Blood Vessels-Akita, Akita-Shi, Japan; 9 National Center Hospital, National Center of Neurology and Psychiary, Kodaira-Shi, Japan; 10 National Center of Geriatrics and Gerontology, Obu-Shi, Japan; 11 Institute of Biomedical Research and Innovation, Kobe-Shi, Japan; 12 Tokyo Metropolitan Geriatric Hospital and Institute of Gerontology, Itabashi-Ku, Japan; 13 Niigata University Medical & Dental Hospital, Niigata-Shi, Japan; 14 The University of Tokyo Hospital, Bunkyo-Ku, Japan; 15 Sapporo Medical University Hospital, Sapporo-Shi, Japan; 16 Hirosaki University School of Medicine & Hospital, Hirosaki City, Japan; 17 Iwate Medical University, Shiwa-Gun, Japan; 18 Gunma University Hospital, Maebashi-Shi, Japan; 19 National Institute of Radiological Sciences, Chiba-Shi, Japan; 20 Asahi Hospital for Neurological Diseases and Rehabilitation, Matsudo-Shi, Japan; 21 National Hospital Organization Chiba-East-Hospital, Chiba-Shi, Japan; 22 Saitama Medical University Hospital, Iruma-Gun, Japan; 23 Juntendo University Hospital, Bunkyo-Ku, Japan; 24 Juntendo Tokyo Koto Geriatric Medical Center, Koto-Ku, Japan; 25 Tokyo Medical University, Shinjuku-Ku, Japan; 26 Nippon Medical School Hospital, Bunkyo-Ku, Japan; 27 Kyorin University Hospital, Mitaka-Shi, Japan; 28 Tokyo Medical and Dental University, University Hospital of Medicine, Bunkyo-Ku, Japan; 29 National Hospital Organization Kurihama Medical and Addicition Center, Yokosuka-Shi, Japan; 30 Shonan-Atsugi Hospital, Atsugi-Shi, Japan; 31 Kanazawa University Hospital, Kanazawa-Shi, Japan; 32 Hamamatsu Medical Center, Hamamatsu City, Japan; 33 Kyoto University Hospital, Kyoto-Shi, Japan; 34 University Hospital, Kyoto Prefectural University of Medicine, Kyoto-Shi, Japan; 35 Nara Medical University Hospital, Kashihara-Shi, Japan; 36 Osaka University Hospital, Suita-Shi, Japan; 37 Osaka City University Hospital, Osaka-Shi, Japan; 38 Kobe University Hospital, Kobe City, Japan; 39 Okayama University Hospital, Okayama Diagnostic Imaging Center, Okayama-Shi, Japan; 40 Tottori University Hospital, Yonago-Shi, Japan; 41 Kagawa University Hospital, Kita-Gun, Japan; 42 Fukuoka University Hospital, Fukuoka-Shi, Japan; 43 Kumamoto University Hospital, Kumamoto-Shi, Japan; 44 Shinshu University Hospital, Matsumoto-Shi, Japan; 45 Mihara Memorial Hospital, Isesaki-Shi, Japan; 46 Yokohama City University Medical Center, Yokohama-Shi, Japan; 47 Kindai University, Higashiosaka City, Japan

**Keywords:** Neuroscience, Diseases, Neurology

## Abstract

We aimed to compare longitudinal brain atrophy in patients with neuromyelitis optica spectrum disorder (NMOSD) with healthy controls (HCs). The atrophy rate in patients with anti-aquaporin-4 antibody-positive NMOSD (AQP4 + NMOSD) was compared with age-sex-matched HCs recruited from the Japanese Alzheimer’s Disease Neuroimaging Initiative study and another study performed at Chiba University. Twenty-nine patients with AQP4 + NMOSD and 29 HCs were enrolled in the study. The time between magnetic resonance imaging (MRI) scans was longer in the AQP4 + NMOSD group compared with the HCs (median; 3.2 vs. 2.9 years, *P* = 0.009). The annualized normalized white matter volume (NWV) atrophy rate was higher in the AQP4 + NMOSD group compared with the HCs (median; 0.37 vs. − 0.14, *P* = 0.018). The maximum spinal cord lesion length negatively correlated with NWV at baseline MRI in patients with AQP4 + NMOSD (Spearman’s rho =  − 0.41, *P* = 0.027). The annualized NWV atrophy rate negatively correlated with the time between initiation of persistent prednisolone usage and baseline MRI in patients with AQP4 + NMOSD (Spearman’s rho =  − 0.43, *P* = 0.019). Patients with AQP4 + NMOSD had a greater annualized NWV atrophy rate than HCs. Suppressing disease activity may prevent brain atrophy in patients with AQP4 + NMOSD.

## Introduction

Neuromyelitis optica spectrum disorder (NMOSD) is a severe form of the central nervous system (CNS) inflammation that typically affects the spinal cord and optic nerve. However, brain lesions can occur in NMOSD. Another feature of NMOSD is positivity for aquaporin-4 (AQP4) antibodies, which are present in the sera of 60%–90% of patients with NMOSD (AQP4 + NMOSD patients)^1,2^.

Disability in patients with NMOSD is reported to be attack-dependent^3^. Therefore, NMOSD is considered to not exhibit attack-independent neurodegeneration. However, previous reports observed decreases in the white matter volume in patients with NMOSD compared to that in healthy controls (HCs)^4–6^. Widespread occult damage in normal-appearing white matter was reported in NMOSD compared to the findings in HCs^7^. A recent study observed a spectrum of astrocytopathy that supports the concept of attack-independent structural changes in the NMOSD pathology^8^. Therefore, attack-independent neurodegeneration might occur in the white matter in patients with NMOSD.

Moreover, unlike multiple sclerosis (MS), NMOSD is not thought to exhibit longitudinal brain atrophy compared with HCs. However, only a few studies have investigated longitudinal brain atrophy in patients with NMOSD, including our study^9,10^. We compared longitudinal brain atrophy in patients with AQP4 + NMOSD to longitudinal brain atrophy in patients with MS and showed that brain atrophy silently progressed in patients with AQP4 + NMOSD, even in clinically inactive patients^10^. Patients with AQP4 + NMOSD and long cord lesions exhibited annualized brain gray matter volume atrophy rates that were higher than patients without long cord lesions^10^. Thus, we hypothesized that subclinical dying back degeneration caused by long cord lesion contributed to the brain atrophy in patients with AQP4 + NMOSD. However, HCs were absent in our previous study. Therefore, we compared the longitudinal brain atrophy rate between patients with NMOSD and HCs to overcome this limitation.

In this study, longitudinal brain atrophy was compared in patients with AQP4 + NMOSD to age-sex-matched HCs using magnetic resonance imaging (MRI) images obtained from the Japanese Alzheimer’s Disease Neuroimaging Initiative (J-ADNI) study and another study performed at Chiba University. We also investigated the clinical characteristics associated with brain volume at baseline in patients with AQP4 + NMOSD.

## Materials and methods

### Study design and patient populations

**Figure 1:**
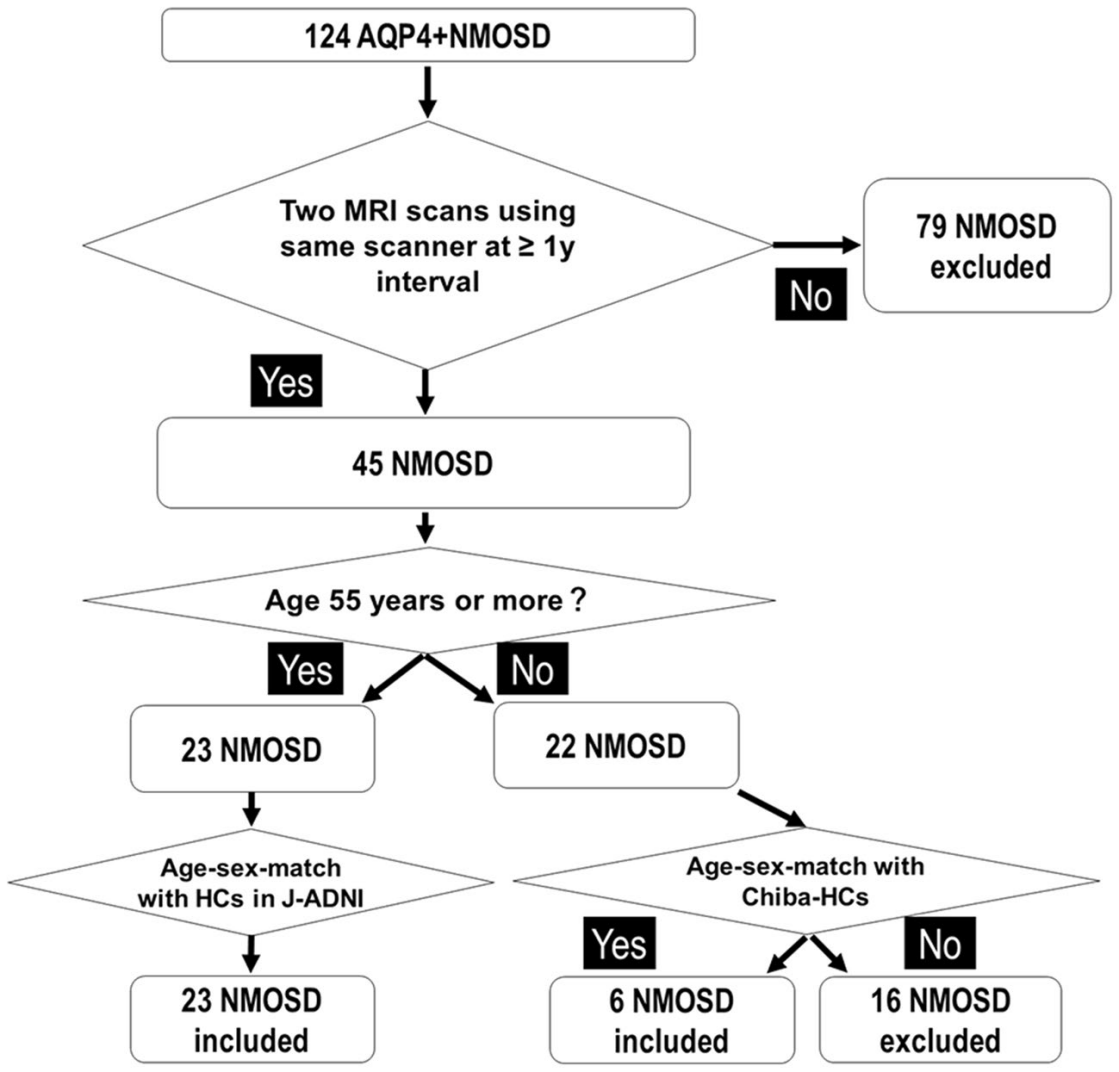
Flow chart and study design showing enrolment and age-sex-matching of patients with AQP4 + NMOSD and HCs. *AQP4 + NMOSD* anti-aquaporin-4 antibody-positive neuromyelitis optica spectrum disorders, *HCs* healthy controls. Chiba-HCs means another study performed in Chiba University by Shimizu et al.

We expanded the previously published dataset^10^. Therefore, patients’ data overlapped with the previous study (82.8%)^10^. We reviewed the clinical records of 124 patients with AQP4 + NMOSD at Chiba University Hospital. Figure 1 demonstrates the patient enrolment and study design. Recruitment of patients with AQP4 + NMOSD was conducted as previously described^10^. First, we included patients with two MRI scans by the same scanner at an interval of > 1 year. We selected two MRI scans (MRI-1 and MRI-2) as the interval widened, as previously reported^11^. We excluded MRIs performed within 60 days of prednisolone pulse therapy or additional immunotherapies including plasma exchange to minimize pseudoatrophy^12,13^. All AQP4 + NMOSD patients fulfilled the 2015 international diagnostic criteria for NMOSD^1^ with AQP4 + cell-based assay results, as previously described^14^. In addition, patients were grouped by age (< 55 years vs. ≥ 55 years) and matched by age and sex with HCs.

Patients who were at least 55 years old were age-sex matched with HCs from the J-ADNI study (UMIN000001374)^15^. The public–private partnership, J-ADNI, was established in 2007 with Professor Takeshi Iwatsubo as the Principal Investigator. The J-ADNI study’s main objective was to ascertain serial magnetic MRI, positron emission tomography, other biological markers, and clinical and neuropsychological assessment could be integrated to track mild Alzheimer’s disease and late mild cognitive impairment in the Japanese population. The National Bioscience Database Center Human Database, Japan (Research ID: hum0043.v1, 2016) provided the J-ADNI data. All J-ADNI MRI data were published after distortion correction^16^.

Volunteer participants between 60 and 84 years old enrolled in the J-ADNI study. The eligibility criteria of the ADNI study was applied to the volunteer participants^17^. Subjects who scored 24–30 in the Mini-Mental State Examination scores without memory complaints were treated as HCs. We included subjects with two same-scanner MRI scans at an interval of > 1 year. The two MRI scans (MRI-1 and MRI-2) with the largest time interval were selected. The J-ADNI database information were obtained from the National Bioscience Database Center Human Database, Japan (Research ID: hum0043.v1, 2016)^15^. Other inclusion and exclusion criteria are described at https://center6.umin.ac.jp/cgi-open-bin/ctr/ctr_view.cgi?recptno=R000012764.

Patients less than 55 years old were age-sex-matched with HCs from another study performed at Chiba University by Professor Shimizu (Chiba-HCs). Chiba-HCs had no history of mental disorder, and they were confirmed to currently not meet the diagnostic criteria for any mental disorder by psychosomatic physicians or psychiatrists by a comprehensive structured interview based on Diagnostic and Statistical Manual of Mental Disorders, Fifth Edition. Any subjects with claustrophobia, head trauma, neurological disorders, or substance abuse were excluded. Nine volunteers underwent two same-scanner MRI scans at an interval of > 1 year.

Patients with AQP4 + NMOSD and HCs were sorted by age. Younger patients with AQP4 + NMOSD and HCs with an age difference of ≤ 5 years were matched. The HC closest in age to the patient was matched. If there were several candidates, patients or controls were blindly selected by a doctor (Yosuke Onishi).

### Demographic characteristics

Demographic characteristics at MRI-1 and MRI-2, including sex ratio and age, and clinical features, including disease duration, Kurtzke’s Expanded Disability Status Scale (EDSS) score, annualized relapse rate (ARR) from disease onset, years from the last attack, years of continuous prednisolone, and oligoclonal band positivity were investigated. Years of the relapse prevention treatment at the same dosage which was given at MRI-1 or MRI-2 were also examined. Histories of optic neuritis, myelitis, long cord lesion, brainstem lesion, area postrema syndrome and cerebral syndrome were also investigated.

The brain volumes at MRI-1 and MRI-2 and atrophy rates in patients with AQP4 + NMOSD and HCs were compared.

### Association of brain volume and clinical characteristics in patients with AQP4 + NMOSD

The maximum spinal cord lesion length and brain volume at baseline were correlated. The associations between annualized brain atrophy rates and clinical characteristics, including treatment duration, EDSS and ARR were also analyzed. Long cord lesion was defined as > 3 vertebral segments. We measured the spinal cord lesion length (vertebral body segments) from the image showing the maximum spinal cord lesion length in all spinal cord images performed in the acute phase of previous myelitis before MRI-1, and analyzed the correlation between the length and annualized atrophy rate. The analysis of association between years of continuous prednisolone usage and brain atrophy rates were added when difference of brain atrophy rates was found between patients and HCs. Differences in the annualized NWV atrophy rate with or without a history of brainstem lesion or cerebral syndrome were investigated in patients with AQP + NMOSD. Clinical and brain volume difference between male and female patients with AQP4 + NMOSD were also investigated.

### Brain MRI scan and brain volume measurements

The same MR scanner, a 1.5-Tesla Signa HDxT (GE Healthcare, Milwaukee, WI, USA), was used to obtain a conventional brain MRI, T1-weighted three-dimensional (3D) images, and fluid-attenuated inversion recovery (FLAIR) or multiplanar reconstruction (MPR) from the 3D-FLAIR from each patient. Supplementary Table S1 shows details of the MRI systems for patients.

Brain MRI imaging of HCs participating in the J-ADNI study was performed using a 1.5-Tesla^15^. The 3.0-Tesla Discovery MR750 (GE Healthcare, Milwaukee, WI, USA) was used for all Chiba-HCs. Supplementary Table S2 presents MRI system information for younger HCs.

Since previous studies demonstrated the different scanners at different time points significantly affected the brain atrophy measures in the longitudinal morphometric results^18,19^, the same MRI scanner was used for individual patients or Chiba-HCs to minimize the effect of the field strength difference between 3.0-Tesla for Chiba-HCs and 1.5-Tesla for all patients. Distortion correction was performed to all J-ADNI MRI data before the data publication^20^.

We calculated brain volumes using statistical parametric mapping-12 (SPM12) with MATLAB (Version R2016b; The MathWorks, Inc., Natick, MA, USA). Measuring brain volume in each patient was performed as described previously^21,22^. We employed a previously described technique to segment lesions and calculate the annualized atrophy rate^10,23^. Lesion Segmentation Tool (LST) toolbox version 2.0.15 (available in the public domain at www.statisticalmodelling.de/lst.html) was used for SPM^23^. As recommended by Schmidt et al.^23^, we used an initial threshold (κ) value of 0.30. Normalized brain (NBV), gray matter (NGV), lesion (NLV), and white matter (NWV) volumes were defined as previously reported^10^. Briefly, each volume was divided by the intracranial volume, which was the sum of the whole-brain gray matter, white matter, and cerebrospinal fluid volumes, to reduce interindividual variation^24^.

### Statistical analysis

Statistical analyses were performed with SPSS version 27.0 (IBM Corporation, Armonk, NY, USA). Continuous data were compared by the Mann–Whitney *U* test. The Fisher’s exact test was used to evaluate categorical outcomes. Correlations were analyzed using the Spearman’s rank test. *P* < 0.05 was considered statistically significant. An analysis of covariance was performed when the annualized atrophy rate was determined using significant different items as covariates.

### Ethical approval and consent to participate

The Chiba University School of Medicine ethic committee approved the study (No. 2555 and M10545). Informed consent was provided by all patients. The methods used in this study comply with the Declaration of Helsinki and its subsequent amendments, and were performed in accordance with the relevant guidelines and regulations.

## Results

### Demographics and clinical characteristics at MRI-1 and MRI-2

We enrolled 29 patients in each group. Twenty-three patients with AQP4 + NMOSD who were at least 55 years old were matched with HCs in J-ADNI. Of the 22 patients less than 55 years old, 6 patients were matched with Chiba-HCs. Three HCs in the Chiba study were not matched because of sex differences. Of the 29 patients who were enrolled in this study, twenty-four patients overlapped with our previous study^10^. Table 1 displays the clinical characteristics and demographics of patients with AQP4 + NMOSD and the HCs at MRI-1. Females accounted for 75.9% of the patients in both groups. The age difference between the two groups was not statistically significant (median: 59.0 vs. 61.0 years, interquartile range: 9.5 vs. 4.5, *P* = 0.30). The median disease duration at MRI-1 was 7.0 years (interquartile range: 13.4, range: 0.3–42.9). The median EDSS score and ARR from disease onset were 3.5 and 0.4, respectively (interquartile ranges: 4.0 and 0.4, ranges: 0.1–9.0 and 0.2–4.0, respectively). The median time from initiating continuous prednisolone therapy was 3.9 years (interquartile range: 3.2, range: 0.1–8.8). The median time since the last attack was 2.8 years (interquartile range: 3.3, range: 0.2–6.9). The median of years of the relapse prevention treatment was 1.3 at MRI-1 (interquartile range: 1.8, range: 0.1–5.2). Twenty patients with AQP4 + NMOSD received prednisolone alone; three patients received prednisolone plus azathioprine, one patient received prednisolone and eculizumab, and five patients did not receive any treatment at MRI-1. Dose range of prednisolone was from 1.25 to 20 mg/day at MRI-1.

**Table 1:** Demographic and clinical characteristics in patients with AQP4 + NMOSD and HCs at MRI-1.

	AQP4 + NMOSD (N = 29)	HCs (N = 29)	*P*-value
Demographic			
Female (%)	22/29 (75.9%)	22/29 (75.9%)	1.00
Age (years)	59.0 [9.5] (34–73)	61.0 [4.5] (33–73)	0.24
Clinical			
Age at disease onset	48.0 [19.0] (19–67)		
Disease duration (years)	7.0 [13.4] (0.3–42.9)		
EDSS score	3.5 [4.0] (1.0–9.0)		
ARR from disease onset	0.4 [0.4] (0.2–4.0)		
Years from last attack	2.8 [3.3] (0.2–6.9)		
Years of continuous prednisolone	3.9 [3.2] (0.1–8.8) (N = 24)		
Years of relapse prevention treatment	1.3 [1.8] (0.1–5.2)		
Oligoclonal bands positivity	2/17 (11.8%)		
Number of patients with a history of			
Optic neuritis	20/29 (69.0%)		
Myelitis	25/29 (86.2%)		
Myelitis with long cord lesion	18/29 (62.1%)		
Brainstem lesion	6/29 (20.7%)		
Area postrema syndrome	1/29 (3.4%)		
Cerebral syndrome	4/29 (13.7%)		
Treatment			
Prednisolone	20		
Prednisolone + azathioprine	3		
Prednisolone + eculizumab	1		
None	5		

Data are presented as median number (%) or [interquartile range] (range). *P < 0.05. Years of the relapse prevention treatment indicate the continuous relapse prevention treatment period at the same dosage which was given at MRI-1.

*AQP4 + NMOSD* anti-aquaporin-4 antibody-positive neuromyelitis optica spectrum disorder disease, *ARR* annualized relapse rate, *EDSS* Kurtzke’s Expanded Disability Status Scale.

The clinical features at MRI-2 in patients with AQP4 + NMOSD and HCs are shown in Table 2.

**Table 2:** Clinical characteristics at MRI-2 and brain volumes in patients with AQP4 + NMOSD and HCs.

	AQP4 + NMOSD (N = 29)	HCs (N = 29)	*P-*value
Years from last attack to MRI-2	5.2 [6.3] (0.9–10.3)		
Years of relapse prevention treatment	3.3 [4.4] (0.8–7.9)		
ΔEDSS (MRI-2 – MRI-1)	0.0 [− 0.5] (− 2.0–3.5)		
Years from MRI-1 to MRI-2	3.2 [2.3] (1.0–6.3)	2.9 [1.1] (1.0–3.0)	0.009*
ARR between MRI-1 and MRI-2	0.0 [0.1] (0.0–0.9)		
At MRI-1			
ICV*10^–3^ (mL)	1.35 [0.14] (1.22–1.60)	1.37 [0.21] (1.18–1.86)	0.40
NLV (mL)	0.96 [4.08] (0.00–16.30)		
NGV*10^–3^ (mL)	0.42 [0.05] (0.32–0.49)	0.45 [0.04] (0.37–0.52)	0.004*
NWV*10^–3^ (mL)	0.30 [0.03] (0.26–0.34)	0.29 [0.03] (0.26–0.34)	0.17
NBV*10^–3^ (mL)	0.73 [0.06] (0.62–0.81)	0.75 [0.04] (0.66–0.85)	0.044*
At MRI-2			
ICV*10^–3^ (mL)	1.35 [0.13] (1.21–1.58)	1.37 [0.21] (1.18–1.86)	0.37
NLV (mL)	1.70 [7.15] (0.00–141.5)		
NGV*10^–3^ (mL)	0.41 [0.06] (0.31–0.49)	0.44 [0.04] (0.37–0.51)	0.008*
NWV*10^–3^ (mL)	0.30 [0.03] (0.24–0.34)	0.30 [0.03] (0.26–0.34)	0.32
NBV*10^–3^ (mL)	0.73 [0.08] (0.59–0.80)	0.74 [0.05] (0.66–0.83)	0.069
Annualized atrophy rate			
NGV (%)	0.44 [0.15] (− 2.44–4.88)	0.71 [1.24] (− 1.43–3.14)	0.43
NWV (%)	0.37 [1.36] (− 6.23–4.48)	− 0.14 [0.92] (− 1.76–1.03)	0.018*
NBV (%)	0.50 [0.75] (− 0.57–3.04)	0.41 [0.75] (− 0.88–1.72)	0.91

Data are presented as median number (%) or [interquartile range] (range). *P < 0.05.

ΔEDSS = EDSS at MRI-2 minus EDSS at MRI-1. Years of the relapse prevention treatment indicate the continuous relapse prevention treatment period at the same dosage which was given at MRI-2.

*AQP4 + NMOSD* anti-aquaporin-4 antibody-positive neuromyelitis optica spectrum disorder disease, *ARR* annual relapse rate, *EDSS* Kurtzke’s Expanded Disability Status Scale, *ICV* intracranial volume, *NBV* normalized brain volume, *NGV* normalized gray matter volume, *NLV* normalized lesion volume, *NWV* normalized white matter volume. Annualized atrophy rate of X is defined as follows; $\frac{{\left( {{\text{X}}\;{\text{ at}}\;{\text{ 1st }}\;{\text{MRI }}\;{\text{scan }} \times {\text{ at }}\;{\text{2nd}}\;{\text{ MRI }}\;{\text{scan}}} \right) \, \times { 12}}}{{\left( {{\text{X }}\;{\text{at }}\;{\text{1st }}\;{\text{MRI }}\;{\text{scan}}} \right) \, \times \, \left( {{\text{Months}}\;{\text{ between }}\;{\text{1st }}\;{\text{and}}\;{\text{ 2nd }}\;{\text{MRI }}\;{\text{scans}}} \right)}}$ , X = NGV, NWV or NBV.

The median years from the last attack to MRI-2 was 5.2 years (interquartile range: 6.3, range: 0.9–10.3). In total, six patients showed the relapse between MRI-1 and MRI-2. Only one patient relapsed with cerebral syndrome between MRI scans. Years from the last attack to MRI-2 in the six patients were 0.90, 0.98, 1.26, 1.28, 1.96, and 2.64. The median of years of the relapse prevention treatment was 3.3 at MRI-2 (interquartile range: 4.4, range: 0.8–7.9). Ten patients changed the relapse prevention treatment between MRI-1 and MRI-2. One patient discontinued relapse prevention and one patient initiated the continuous prednisolone. Within other eight patients, four patients decreased the dosage of prednisolone between MRI-1 and MRI-2, two patients increased the prednisolone dosage, and two patients showed the same dosage of prednisolone. Nineteen patients with AQP4 + NMOSD received prednisolone alone; four patients received prednisolone plus azathioprine, one patient received prednisolone and eculizumab, and five patients received no treatment at MRI-2. Dose range of prednisolone was from 5.0 mg/day to 15 mg/day at MRI-2.

### Linearity of changes in brain volume in patients with AQP4 + NMOSD

**Figure 2:**
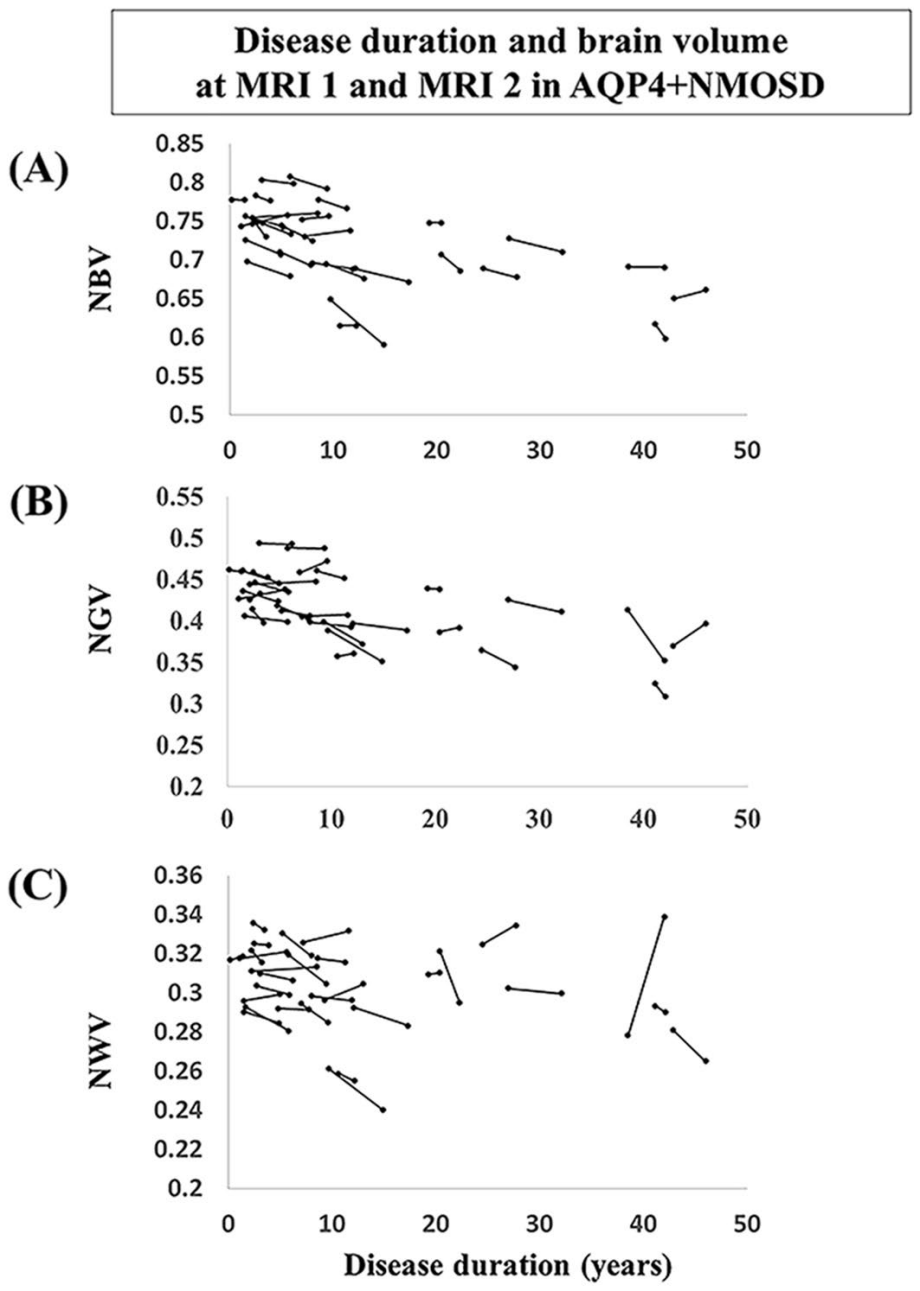
Brain volume changes and disease duration in each patient with AQP4 + NMOSD. (**A**) NBV changes and disease durations. (**B**) NGV changes and disease durations. (**C**) NWV changes and disease durations. *AQP4 + NMOSD* anti-aquaporin-4 antibody-positive neuromyelitis optica spectrum disorders, *NBV* normalized brain volume, *NGV* normalized gray matter volume, *NWV* normalized white matter volume.

To establish the linearity of longitudinal brain atrophy in patients with AQP4 + NMOSD, brain volume changes and disease duration in each patient with AQP4 + NMOSD were analyzed. Most patients showed a similar slope for changes in brain atrophy between MRI-1 and MRI-2, regardless of the disease duration (Fig. 2).

### Annualized NWV atrophy rate

**Figure 3:**
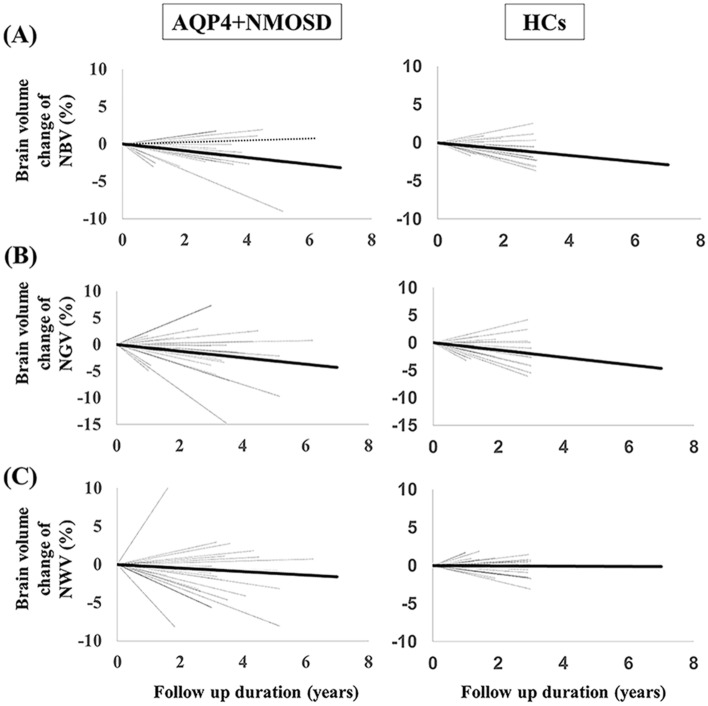
The percent brain volume changes between MRI-1 and MRI-2 in patients with AQP4 + NMOSD and HCs. (**A**) NBV changes and follow-up durations. (**B**) NGV changes and follow-up durations. (**C**) NWV changes and follow-up durations. The black dotted lines represent brain volume changes (in percentage) for each patient. The fitted average slopes in patients with AQP4 + NMOSD and HCs are shown by the black line. *AQP4 + NMOSD* anti-aquaporin-4 antibody-positive neuromyelitis optica spectrum disorders, *HCs* healthy controls, *NBV* normalized brain volume, *NGV* normalized gray matter volume, *NWV* normalized white matter volume.

Table 2 shows the brain volumes at MRI-1 and MRI-2 as well as the annualized atrophy rate between MRI-1 and MRI-2 in patients with AQP4 + NMOSD and HCs. Patients with AQP4 + NMOSD had significantly longer intervals between MRI-1 and MRI-2 than HCs (median: 3.2 vs. 2.9, *P* = 0.009). Patients with AQP4 + NMOSD had decreased NBV at MRI-1 and NGV at MRI-1 and MRI-2 compared to NBV and NGV in HCs. Although the annualized NGW and NBV atrophy rates were not significantly different between patients with AQP4 + NMOSD and HCs, the patients with AQP4 + NMOSD had an annualized NWV atrophy rate greater than that of HCs (*P* = 0.018). The annualized NWV atrophy rate was significantly related to the MRI-1 and MRI-2 time interval in patients with AQP4 + NMOSD and HCs, according to the parallelism test (*P* = 0.048). Therefore, an analysis of covariance using the MRI-1 and MRI-2 time interval as covariates could not be performed for the annualized atrophy rate of NWV. Figure 3 shows the percentage of NBV, NGV, and NWV changes in patients with AQP4 + NMOSD and HCs.

### Correlation between spinal cord lesion length and NWV at MRI-1

**Figure 4:**
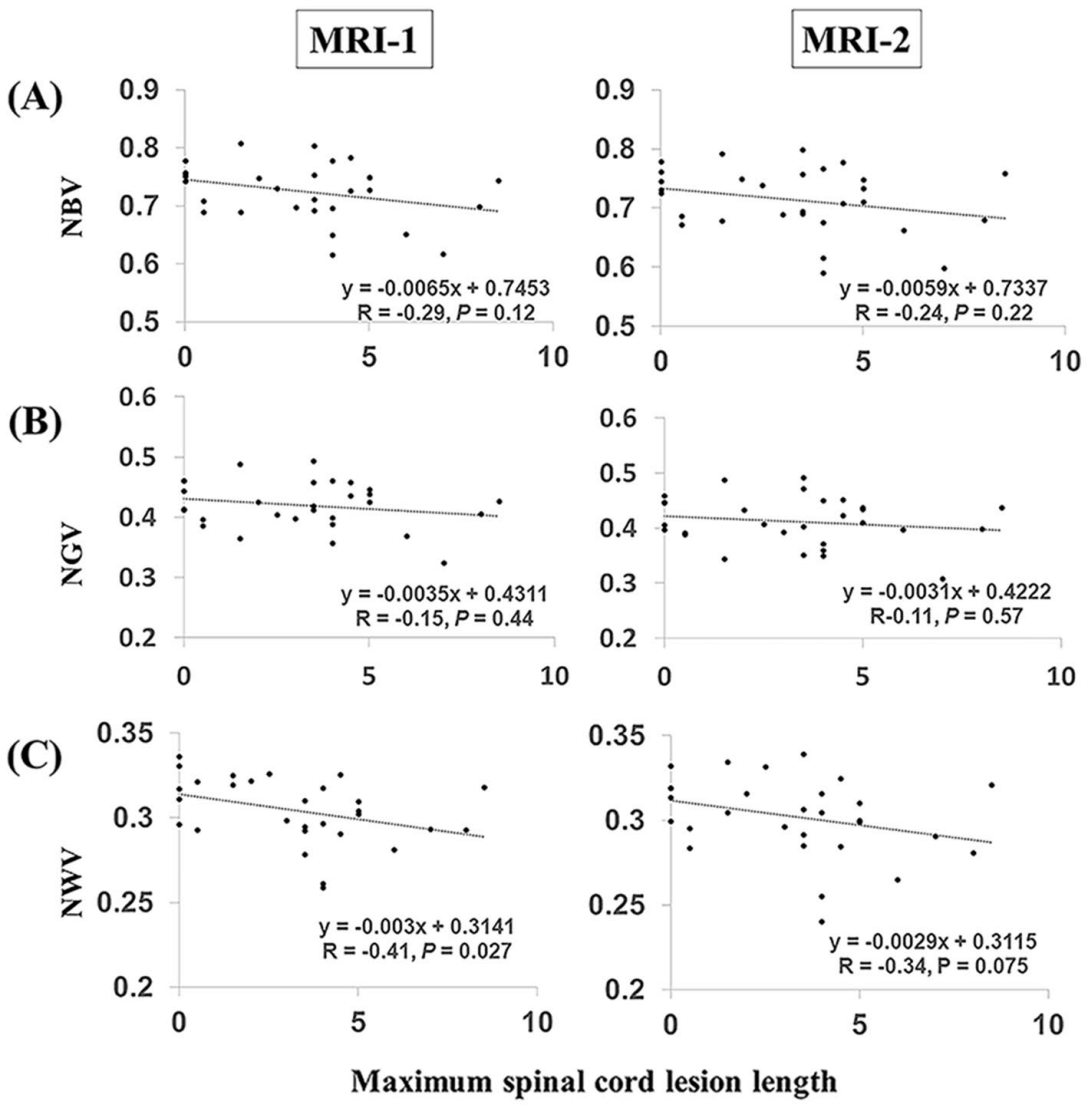
Correlation between brain volumes and spinal lesion cord length in patients with AQP4 + NMOSD. (**A**) NBV changes and maximum spinal cord lesion length. (**B**) NGV changes and maximum spinal cord lesion length. (**C**) NWV changes and maximum spinal cord lesion length. *AQP4 + NMOSD* anti-aquaporin-4 antibody-positive neuromyelitis optica spectrum disorders, *NBV* normalized brain volume, *NGV* normalized gray matter volume, *NWV* normalized white matter volume.

In patients with AQP4 + NMOSD, we examined the relationships between the annualized atrophy rate of each brain volume and the spinal cord lesion length and brain volumes at MRI-1 and MRI-2. The spinal cord lesion length negatively correlated with NWV at MRI-1 (Spearman’s rho =  − 0.41, *P* = 0.027, Fig. 4) but not with NWV at MRI-2, NBV at MRI-1 or MRI-2, NGV at MRI-1 or MRI-2, or NLV at MRI-1 or MRI-2. No associations were found between the spinal cord lesion length and annualized atrophy rate of each brain volumes including NBV, NGV, and NWV.

### Correlation between persistent prednisolone usage duration and the annualized NWV atrophy rate

**Figure 5:**
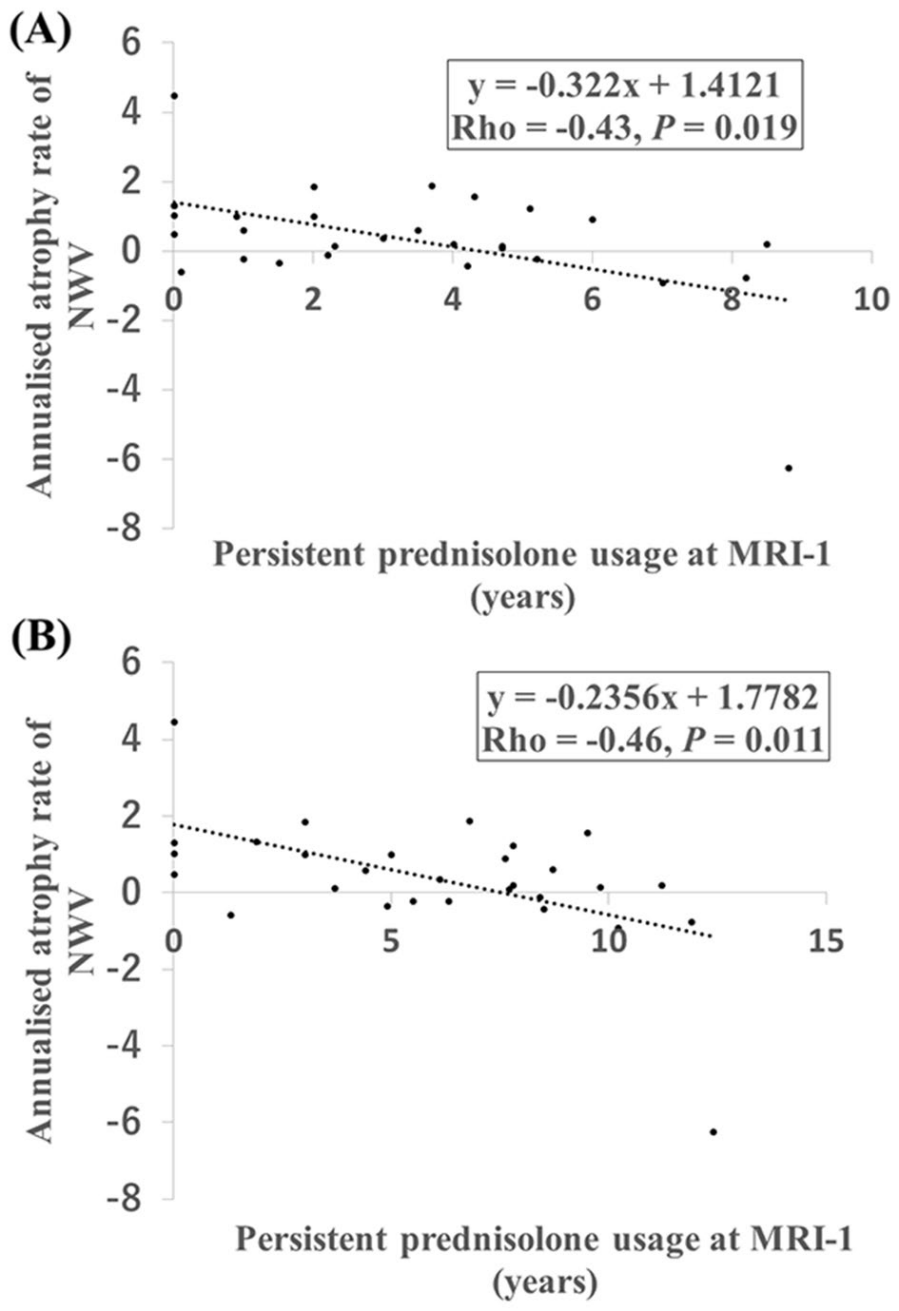
Correlation between persistent prednisolone usage and the annualized NWV atrophy rate in patients with AQP4 + NMOSD. (**A**) The annualized NWV atrophy rates and persistent prednisolone usage durations at MRI-1. (**B**) The annualized NWV atrophy rates and persistent prednisolone usage durations at MRI-2. *AQP4 + NMOSD* anti-aquaporin-4 antibody-positive neuromyelitis optica spectrum disorders, *NWV* normalized white matter volume.

The annualized NWV atrophy rate negatively correlated with the time from the initiation of persistent prednisolone usage to MRI-1 and MRI-2 in patients with AQP4 + NMOSD (Spearman’s rho =  − 0.43 and − 0.46, *P* = 0.019 and 0.011, respectively, Fig. 5). EDSS at MRI-1, ΔEDSS (EDSS at MRI-2 minus EDSS at MRI-1), and ARR between MRI-1 and MRI-2 did not correlate with the annualized NBV, NGV or NWV atrophy rates. On the other hand, ARR at MRI-1 negatively correlated with the annualized NWV atrophy rate (Spearman’s rho =  − 0.41, *P* = 0.026) but not with the annualized NBV or NGV atrophy rates. In patients with persistent prednisolone at MRI-1, ARR at MRI-1 negatively correlated with the duration of persistent prednisolone use (Spearman’s rho =  − 0.44, *P* = 0.033). No correlation was found between the annualized NWV atrophy rate and disease duration at MRI-1, MRI-2, or the interval between MRI-1 and MRI-2.

### Difference in the annualized NWV atrophy rate between patients with and without a history of brainstem lesion or cerebral syndrome

No difference was found in the annualized NWV atrophy rate between patients with (N = 6) and those without a history of brainstem lesion (N = 23). The annualized NWV atrophy rate was higher in patients with a history of cerebral syndrome than in those without history of cerebral syndrome (median: 1.38% vs. 0.20%, interquartile range: 1.13 vs. 1.30, N = 4 vs. N = 25,* P* = 0.043). Patients with a history of cerebral syndrome had higher NLV at MRI-1 than those without history of cerebral syndrome (median: 11.5. mL vs. 0.91 mL, interquartile range: 12.8 vs. 3.07, N = 4 vs. N = 25,* P* = 0.008). Patients without cerebral syndrome tended to have a higher annualized NWV atrophy rate than age- and sex-matched HCs (median: 0.20% vs. − 0.17%, interquartile range: 1.30 vs. 0.96, N = 25 vs. N = 25,* P* = 0.068). One patient had a history of both brainstem lesion and cerebral syndrome. Patients with a history of brainstem lesion or cerebral lesion (N = 9) showed no difference from those without these histories (N = 20).

### Clinical and brain volume difference between male and female patients with AQP4 + NMOSD

Male patients showed shorter disease duration at MRI-1 (median: 2.4 vs. 9.0 years, *P* = 0.013) and MRI-2 (median: 5.6 vs. 11.9 years, *P* = 0.013) than female patients. The male patients had higher intracranial volume at MRI-1 (median: 1.52 vs. 1.32, *P* < 0.001) and MRI-2 (median: 1.52 vs. 1.32, *P* < 0.001), and age at disease onset (median: 54.0 vs. 46.5 years, *P* = 0.049) than female patients. No difference was found in NBV, NGV, NWV, and NLV at MRI-1 and MRI-2 between male and female patients. Annualized atrophy rates of NBV, NGV, and NWV were similar in male and female patients.

## Discussion

Our results show that patients with AQP4 + NMOSD had significantly higher annualized NWV atrophy rates than age-sex-matched HCs. Spinal cord lesion length negatively correlated with NWV in patients with AQP4 + NMOSD. Persistent prednisolone usage negatively correlated with the annualized NWV atrophy rate. These findings suggest that suppressing disease activity may prevent longitudinal brain atrophy in patients with AQP4 + NMOSD.

Patients with AQP4 + NMOSD had higher annualized NWV atrophy rates than HCs. Our result corresponds with previous studies. Decreased brain white matter volume was reported in NMO compared with HCs^4–6^. Another study reported widespread occult damage in normal-appearing white matter in NMOSD compared with HCs^7^. These results clearly demonstrate brain white matter could be damaged in AQP4 + NMOSD. On the other hand, in HCs, previous studies showed brain white matter volume appeared to be relatively stable except in oldest participants, while brain gray matter volume loss appeared to be constant throughout the adult life^25,26^. Meanwhile, a previous study demonstrated patients with acute spinal cord injury showed faster atrophy rates in brain white matter and gray matter compared with HCs^27^. These findings may explain why we found differences only in NWV but not NGV between patients with AQP4 + NMOSD and HCs.

Our study revealed a negative correlation between the spinal cord lesion length and NWV in patients with NMOSD. Another study reported a lower lateral geniculate nucleus volume in patients with NMOSD and a history of ON than in patients with NMOSD without a history of ON and controls^28^. Oral prednisolone maintenance therapy was reported to be effective to prevent relapse in patients with AQP4 + NMOSD^29^. Moreover, some biological disease-modifying drugs were reported to lower disability or reduce the risk of disability progression in patients with AQP4 + NMOSD. Interleukin-6 receptor blockade decreases ARR and lowers disability in patients with NMOSD^30,31^. CD-19 blockade was also demonstrated to decrease the risk of 3-month EDSS-confirmed disability progression in patients with NMOSD^32^. In addition, MRI studies demonstrated decreased spinal cord MRI activity during tocilizumab therapy, particularly in patients with AQP4 + NMOSD^30^. The mean upper cervical cord area was associated with normalized brain volume in patients with MS^33^. Cortical atrophy following spinal cord injury was also reported^34^. Therefore, if silent progression by subclinical dying back degeneration occurs in patients with AQP4 + NMOSD, as we hypothesized, then biological disease-modifying drugs can prevent brain atrophy by decreasing the activity and lesion length in the spinal cord.

We demonstrated a negative correlation between annualized NWV atrophy rate and ARR at MRI-1. In addition, ARR at MRI-1 negatively correlated with persistent prednisolone duration at MRI-1 in patients undergoing persistent prednisolone treatment. These results suggest that inhibiting relapse and inflammation lowers subsequent brain white matter atrophy rates in patients with AQP4 + NMOSD. Autopsy results from a patient with AQP4 + NMO showed persistent microscopic active inflammatory lesions in the CNS^35^. The patient received oral prednisolone treatment for over 40 years and showed no relapse for more than five years before death. Microscopic active inflammatory lesions were found not only in the spinal cord but also in the white matter of the right frontal lobe, left amygdala and central pons. These subclinical microscopic active inflammatory lesions in the brain may result in higher longitudinal brain atrophy rates in patients with AQP4 + NMOSD. This hypothesis may explain our findings of a negative correlation between longer prednisolone usage and annualized NWV atrophy rate. Conversely, cerebral syndrome, such as higher brain dysfunction, tends not to be reflected in the EDSS score. Moreover, attack-independent structural changes were reported in the NMOSD pathology^8^. These facts could explain our results, in which ΔEDSS and ARR between MRI-1 and MRI-2 was not correlated with the annualized NBV, NGV, and NWV atrophy rates. Moreover, tiny structural changes in the brain without clinical relapse or EDSS changes might also contribute to brain atrophy in patients with NMOSD. Therefore, not only dying back degeneration but also subclinical active lesions may cause brain atrophy in patients with AQP4 + NMOSD. However, another study reported two cases of progressive cerebral atrophy in patients with NMO^36^. The authors speculated that progressive cerebral cortical atrophy is induced by severe intrathecal inflammation in patients with NMO. Meanwhile, previous studies reported that chronic steroid use might contribute to the loss of brain tissue^37,38^. These reports are not consistent with our findings, in which persistent prednisolone usage had a negative correlation with the annualized NWV atrophy rate. However, chronic steroid use could inhibit the silent progression in NMOSD as we hypothesized in our previous study. Therefore, further investigation is required regarding the underlying mechanisms of brain atrophy in patients with AQP4 + NMOSD.

Our study has several limitations. First, this study included the same patient data as our previous report (24/29, 82.8%)^10^. Therefore, further study using different patients is required. Second, MRI imaging performed in Chiba-HCs did not include FLAIR or MPR. FLAIR or MRP images are required to perform lesion filling. Thus, lesion filling could not be performed only in the younger Chiba-HCs. In general, lacking lesion filling should result in decreased brain volumes. Therefore, if the subclinical cerebral lesions occur between MRI-1 and MRI-2 in Chiba-HCs, the brain volume of Chiba-HCs at MRI-2 should be calculated as lower, resulting in the higher brain atrophy rates in Chiba-HCs. However, higher brain atrophy rates in Chiba-HCs would not affect our conclusion. Third, we used 3.0-Tesla MRI imaging only in younger HCs for the comparison of brain volumes between MRI-1 and MRI-2. Validation is required, particularly for the cross-sectional study, when using different scanners or different magnetic field strength imaging. Therefore, the use of different scanners may have affected our cross-sectional study. However, because individual patients or HCs underwent MRI with the same scanner for the longitudinal study, our results concerning atrophy rates should not be affected. Moreover, a previous study reported that different tesla did not affect brain atrophy results^39^. Fourth, linear brain atrophy was hypothesized in our study. Therefore, the annualized NWV atrophy rate exhibited interaction with the MRI interval according to the parallelism test. If non-linear atrophy occurs, studies from other groups or several time points may be required. Fifth, six patients in our study demonstrated a relapse between MRI-1 and MRI-2. The proportions of patients with relapse between MRI scans may influence the result. Finally, the interval between MRIs was different in the AQP4 + NMOSD and HC groups. No differences in NVWs at either MRI-1 or MRI-2 were observed between patients with AQP4 + NMOSD and HCs, while a higher annualized NWV atrophy rate was observed in patients with AQP4 + NMOSD. A prospective study comparing patients with AQP4 + NMOSD and HCs with the same MRI duration is required to identify the best MRI follow-up interval for detecting NWV differences.

Our study demonstrated that patients with AQP4 + NMOSD had greater rates of longitudinal brain white matter atrophy than HCs. Not only dying back degeneration but also subclinical active lesions may be involved in brain white matter atrophy pathogenesis in patients with AQP4 + NMOSD. Previous studies demonstrated some differences in clinical and demographic features in patients with NMOSD among different ethnic or geographic groups^40,41^. Confounding factors including smoking may be involved in the brain atrophy in MS and NMOSD^42^. Future studies with a higher number of patients, different ethnicity groups, adjusted confounding factors, and a unified MRI scanner are required. Evaluating differences in atrophy rates in patients with or without biological disease-modifying drugs, such as anti-interleukin-6 receptor therapy, may be necessary to determine whether preventing MRI activation in the spinal cord or subclinical active lesions prevents brain atrophy in patients with AQP4 + NMOSD.

### Supplementary Information

Supplementary Tables.

## Data Availability

The datasets analyzed during the current study are available from the corresponding author on reasonable request.
