# Supplementary material for: Higher longitudinal brain white matter atrophy rate in aquaporin-4 IgG-positive NMOSD compared with healthy controls
Source: Sci Rep. 2023 Aug 3;13:12631. doi: 10.1038/s41598-023-38893-1 (PMC10400628; doi:10.1038/s41598-023-38893-1)
Supplement: Supplementary file 1 — Supplementary Tables. [file 41598_2023_38893_MOESM1_ESM.pdf]

Supplementary Table S1. Details of MRI for patients.

	3DT1	FLAIR	MRP reconstructed from 3D-FLAIR
Tesla	1.5	1.5	1.5
FOV (mm×mm)	240 × 240	220 × 220	250 × 250
Number of sections	248	32	75–105
Section thickness (mm)	1.4	4	2
TR (ms)	7	11000–10002	6000
TE (ms)	2.9–3.0	120.2–128.6	131
TI (ms)	0–420	2400	1852
NSA	0.9921–1	1	1
Echo train length	1	1	160
FA	15°	90°	90°

*P < 0.05. 3D-FLAIR: fluid attenuated inversion recovery three-dimensional images;

3DT1: T1-weighted three-dimensional images; FA: flip angle; FLAIR: fluid attenuated inversion recovery; FOV: field of view; NSA: Number of signals acquired; MPR: multiplanar reconstruction; TR: repetition time; TE: echo time; TI: inversion time.

Supplementary Table S2. Detail of MRI system in controls aged less than 55 years old.

	Discovery MR750
Tesla	3.0
3DT1 (SPGR)	
FOV	260mm × 260mm
Number of sections	178
Section thickness	1.2mm
TR	8.132ms
TE	3.164ms
TI	400ms
Number of signals acquired	1
Echo train length	1
FA	11°

**P* < 0.05. 3DT1: T1-weighted three-dimensional images; FA: flip angle; FOV: field of view; NA: not acquired; SPGR: spoiled gradient echo; TR: repetition time; TE: echo time; TI: inversion time.
